# Supplementary material for: Accel-Align: a fast sequence mapper and aligner based on the seed–embed–extend method
Source: BMC Bioinformatics. 2021 May 20;22:257. doi: 10.1186/s12859-021-04162-z (PMC8139006; doi:10.1186/s12859-021-04162-z)
Supplement: Supplementary file 1 — Additional file 1. The link to the used datasets, command line to generate simulated reads, and link to repository of Accel-Align. [file 12859_2021_4162_MOESM1_ESM.docx]

1. **Datasets**

The human reference genome hg37 can be downloaded from <ftp://ftp-trace.ncbi.nih.gov/1000genomes/ftp/technical/reference/human_g1k_v37.fasta.gz>

The Human exome NA12878 can be accessed from <https://www.ncbi.nlm.nih.gov/sra/?term=SRR098401>

The high confidence region BED file from Genome in a Bottle v2.19 (GiaB) can be accessed from <ftp://ftp-trace.ncbi.nlm.nih.gov/giab/ftp/release/NA12878_HG001/NISTv2.19/>

The target region BED file ELID: S0293689 can be accessed from Agilent SureDesgin (<http://earray.chem/agilent.com/suredesign>), point of contact [claude.revel@agilent.com](mailto:claude.revel@agilent.com).

1. **Simulated data**

The illuminia pair-end reads are simulated by Mason2. For example, we use the following command to simulate 10 millions of 150 bp pair-end reads and their coordinates.

*$ mason_simulator --illumina-read-length 150 -ir ref.fna -n 10000000 \*

*-o read1.fastq -or read2.fastq -oa aligned.sam*

1. **Source code**

The source code of Accel-align is openly accessible from <https://github.com/raja-appuswamy/accel-align-release>. Typically, the installation and usage is explained in the README file of the code repository.
